# Supplementary material for: Motor Clustering Enhances Kinesin-driven Vesicle Transport
Source: bioRxiv. 2024 Oct 27:2024.10.23.619892. Preprint. [Version 1] doi: 10.1101/2024.10.23.619892 (PMC11526910; doi:10.1101/2024.10.23.619892)
Supplement: Supplement 1 [file NIHPP2024.10.23.619892v1-supplement-1.pdf]

bioRxiv preprint doi: https://doi.org/10.1101/2024.10.23.619892; this version posted October 27, 2024. The copyright holder for this preprint (which was not certified by peer review) is the author/funder, who has granted bioRxiv a license to display the preprint in perpetuity. It is made available under aCC-BY 4.0 International license.

Supplementary Information

Motor Clustering Enhances Kinesin-driven Vesicle Transport

Rui Jiang, Qingzhou Feng, Daguan Nong, You Jung Kang, David Sept, William O. Hancock

Supplementary Text

Distribution of integrated GFP intensity of single kinesin-1 motors

Each kinesin-1 dimer has two GFPs and the probability of each GFP being fluorescent (termed p) is independent. Since only motors with a fluorescent signal were included in the measurement, the resultant intensity distribution can be described by a sum of two independent normal distributions.

The integrated fluorescent intensity of a single GFP follows a normal distribution N(μ,σ^2), with a probability density function (pdf):

$$f_1(x;\mu,\sigma) = \frac{1}{\sigma\sqrt{2\pi}} e^{-\frac{(x-\mu)^2}{2\sigma^2}} \quad [1]$$

The integrated fluorescent intensity of two GFPs follows a normal distribution N(2μ,2σ^2) with a pdf:

$$f_2(x;\mu,\sigma) = \frac{1}{\sigma\sqrt{4\pi}} e^{-\frac{(x-2\mu)^2}{4\sigma^2}} \quad [2]$$

The amplitude of f_1 (termed A_1) can be calculated from p:

$$A_1 = \frac{2p(1-p)}{2p(1-p)+p^2} \quad [3]$$

The pdf of integrated GFP intensity of a single kinesin-1 motor is therefore:

$$f(x;\mu,\sigma,A_1) = A_1 \frac{1}{\sigma\sqrt{2\pi}} e^{-\frac{(x-\mu)^2}{2\sigma^2}} + (1-A_1)\frac{1}{\sigma\sqrt{4\pi}} e^{-\frac{(x-2\mu)^2}{4\sigma^2}} \quad [4]$$

On each experimental day, the GFP intensity of single kinesin-1 motors were measured and fit to Eq. **4** by maximum likelihood estimation (MLE) to obtain μ and σ (Fig. 1D).

Distribution of integrated GFP intensity of n motors

The probability of *i* GFPs among the *n* motors being fluorescent:

$$\binom{2n}{i} p^i (1-p)^{2n-i} = \frac{(2n)!}{i!(2k-i)!} p^i (1-p)^{2n-i} \quad [5]$$

Since liposomes without any GFP signal were not included in the analysis, the probability calculated in Eq. **5** is normalized by the probability of any number of GFPs being fluorescent:

$$\frac{\frac{(2n)!}{i!(2k-i)!} p^i (1-p)^{2n-i}}{1-(1-p)^{2n}} \quad [6]$$

The integrated intensity of *i* GFPs follows a normal distribution N(iμ,iσ^2), with a pdf:

bioRxiv preprint doi: https://doi.org/10.1101/2024.10.23.619892; this version posted October 27, 2024. The copyright holder for this preprint (which was not certified by peer review) is the author/funder, who has granted bioRxiv a license to display the preprint in perpetuity. It is made available under aCC-BY 4.0 International license.

$$f_i(x;\mu,\sigma) = \frac{1}{\sigma\sqrt{2\pi i}} e^{-\frac{(x-i\mu)^2}{2i\sigma^2}} \quad [7]$$

The pdf of the integrated GFP intensity of *n* motors is calculated as:

$$F(x;\mu,\sigma,n) = \sum_{i=1}^{2n} \frac{\frac{(2n)!}{i!(2n-i)!}p^i(1-p)^{2n-i}}{1-(1-p)^{2n}} \cdot \frac{1}{\sigma\sqrt{2\pi i}} e^{-\frac{(x-i\mu)^2}{2i\sigma^2}} \quad [8]$$

Distribution of motor numbers on liposomes

According to the liposome diameter distribution measured by DLS (Fig. 1B), the probability that a liposome is of area A_j (termed p_{Aj}) is calculated (j = 1-13). Assuming that the surface motor density (ρ) is the same across all liposome sizes under each experimental condition and that motor number on liposomes of area A_j follows a poisson distribution Pois(ρA_j), the pdf of motor number across all liposome sizes can be calculated as:

$$P(X=n;\rho) = \sum_{j=1}^{13} p_{A_j} \frac{(\rho A_j)^n \cdot e^{-\rho A_j}}{n!} \quad [9]$$

Since vesicles with zero motors were excluded from the analysis, the pdf is normalized as:

$$P(X=n;\rho) = \sum_{j=1}^{13} p_{A_j} \frac{(\rho A_j)^n \cdot e^{-\rho A_j}}{n!(1-e^{-\rho A_j})} \quad [10]$$

Distribution of integrated GFP intensity on liposomes

Combining Eqs. **8** and **10**, the pdf of the integrated GFP intensity on liposomes can be expressed as:

$$I(x;\mu,\sigma,\rho) = \sum_{n=1}^{\infty}\left(\sum_{j=1}^{13} p_{A_j} \frac{(\rho A_j)^n \cdot e^{-\rho A_j}}{n!(1-e^{-\rho A_j})}\right)\left(\sum_{i=1}^{2n} \frac{\frac{(2n)!}{i!(2n-i)!}p^i(1-p)^{2n-i}}{1-(1-p)^{2n}} \cdot \frac{1}{\sigma\sqrt{2\pi i}} e^{-\frac{(x-i\mu)^2}{2i\sigma^2}}\right) \quad [11]$$

bioRxiv preprint doi: https://doi.org/10.1101/2024.10.23.619892; this version posted October 27, 2024. The copyright holder for this preprint (which was not certified by peer review) is the author/funder, who has granted bioRxiv a license to display the preprint in perpetuity. It is made available under aCC-BY 4.0 International license.

Supplementary Figures

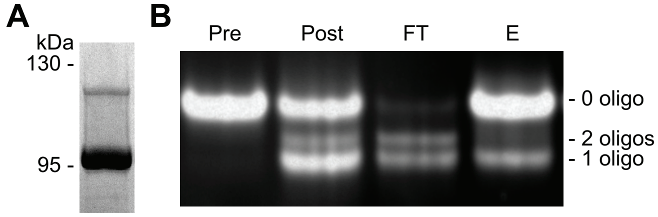

Supplementary Figure 1. Characterization of K406GFP-SNAP by electrophoresis. (A) Coomassie staining of SDS-PAGE showing unlabeled K406GFP-SNAP monomer at 93 kDa, and K406GFP-SNAP-oligo monomer at 114 kDa. (B) Native PAGE showing K406GFP-SNAP samples at different stages of the oligo labeling process, imaged by GFP fluorescence. Pre, K406GFP-SNAP dimers before labeling. Post, K406GFP-SNAP and oligo mixture after the reaction. FT, the portion of the post sample that does not bind to Ni resin (flow through) in the second round of purification. E, eluted sample from Ni resin. Note that the running speed of the 3 species on a native gel is K406GFP-SNAP dimer + 1 oligo > K406GFP-SNAP dimer + 2 oligos > K406GFP-SNAP dimer. This is supported by the preferential exclusion of the two-oligo population from the Ni resin.

bioRxiv preprint doi: https://doi.org/10.1101/2024.10.23.619892; this version posted October 27, 2024. The copyright holder for this preprint (which was not certified by peer review) is the author/funder, who has granted bioRxiv a license to display the preprint in perpetuity. It is made available under aCC-BY 4.0 International license.

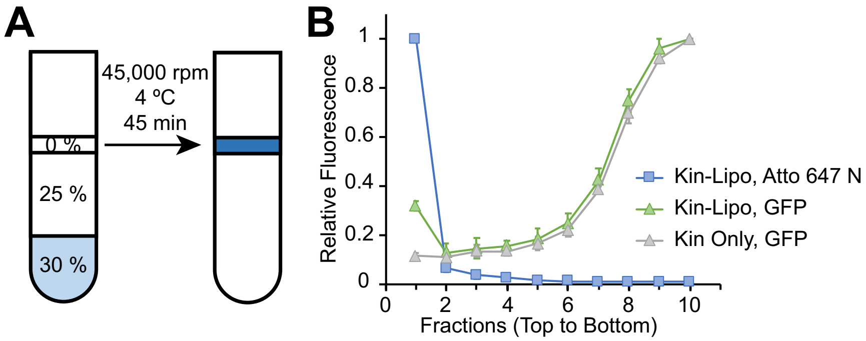

Supplementary Figure 2. Liposome flotation assay.

(A) Schematic of liposome flotation assay. Liposome and motor mixture is loaded to the bottom layer of a sucrose gradient. Upon centrifugation, liposomes with the attached motors 'float' to the top layer, while the unattached motors remain at the bottom. (B) Sample distribution post flotation. Blue squares and green triangles show relative fluorescence signal of liposomes and motors, respectively, when the mixture is loaded to the bottom layer prior to flotation. Grey triangles show motor distribution post flotation when liposomes are omitted. Each data point represents the mean of three independent measurements. Error bars indicate SD.

bioRxiv preprint doi: https://doi.org/10.1101/2024.10.23.619892; this version posted October 27, 2024. The copyright holder for this preprint (which was not certified by peer review) is the author/funder, who has granted bioRxiv a license to display the preprint in perpetuity. It is made available under aCC-BY 4.0 International license.

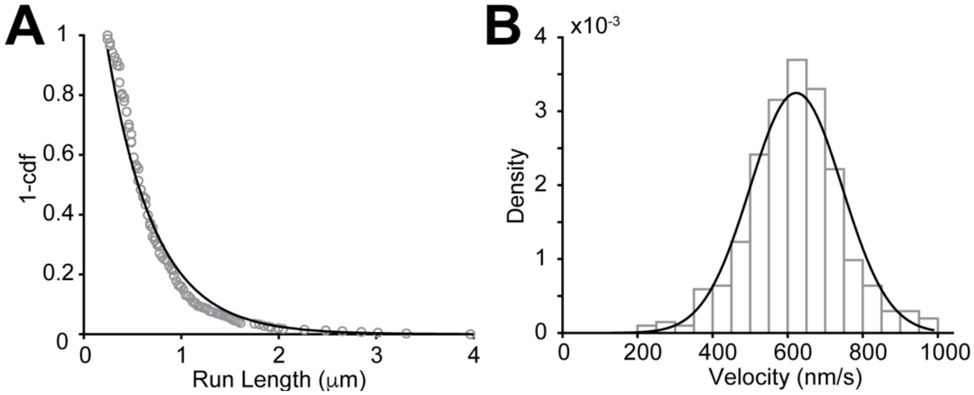

Supplementary Figure 3. Single molecule characterization of K406GFP-SNAP.
(A) Single molecule run length of K406GFP-SNAP. Grey open circles show distribution of raw data (N = 406), black line shows exponential fitting, λ = 0.49 ± 0.02 μm (95% CI of fit). (B) Histogram of K406GFP-SNAP single molecule velocity (N = 406) and fit to a normal distribution. v = 623 ± 123 nm/s (mean ± SD from fit).

bioRxiv preprint doi: https://doi.org/10.1101/2024.10.23.619892; this version posted October 27, 2024. The copyright holder for this preprint (which was not certified by peer review) is the author/funder, who has granted bioRxiv a license to display the preprint in perpetuity. It is made available under aCC-BY 4.0 International license.

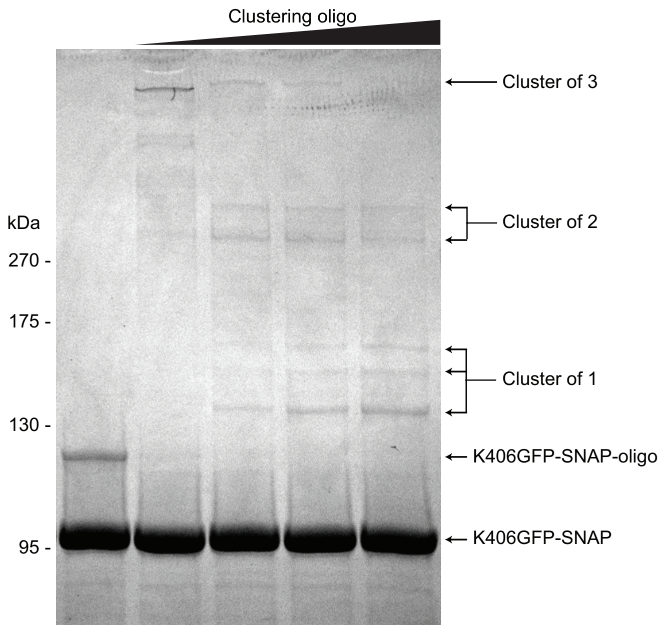

Supplementary Figure 4. Electrophoretic mobility shift assay (EMSA) by SDS-PAGE confirms motor cluster formation. 15 fmol K406GFP-SNAP dimers mixed with increasing amount of the clustering oligo (left to right, 0, 2, 3.75, 5, 7.5 fmol) are loaded to each well. Proteins are stained by Coomassie blue.

bioRxiv preprint doi: https://doi.org/10.1101/2024.10.23.619892; this version posted October 27, 2024. The copyright holder for this preprint (which was not certified by peer review) is the author/funder, who has granted bioRxiv a license to display the preprint in perpetuity. It is made available under aCC-BY 4.0 International license.

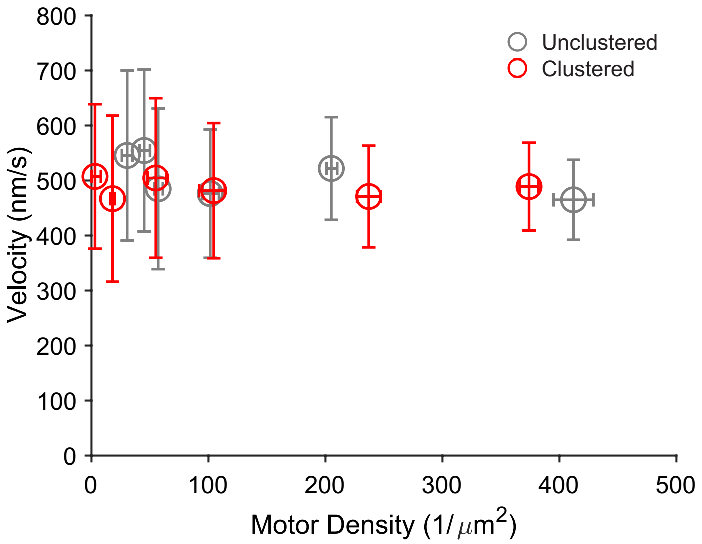

Supplementary Figure 5. Liposome velocity at varying motor densities when motors are unclustered (gray) or clustered (red). Vertical error bars represent SD, horizontal error bars are calculated based on 10% increase in summed squared residuals.
